# Associations of self-rated health, depression, and work ability with employee control over working time

**DOI:** 10.4178/epih.e2025036

**Published:** 2025-07-08

**Authors:** Heejoo Ko, Seong-Sik Cho, Jaesung Choi, Mo-Yeol Kang

**Affiliations:** 1 College of Medicine, The Catholic University of Korea, Seoul, Korea; 2 Department of Occupational and Environmental Medicine, Dong-A University College of Medicine, Busan, Korea; 3 Department of Global Economics, Sungkyunkwan University, Seoul, Korea; 4 Department of Occupational and Environmental Medicine, College of Medicine, The Catholic University of Korea, Seoul, Korea

**Keywords:** Work-time control, Self-rated health, Depression, Work ability

## Abstract

**OBJECTIVES:**

Work-time control (WTC), defined as employees’ ability to influence their work schedules, is a crucial determinant of work-life balance and well-being. This study aims to evaluate the associations between WTC and health-related outcomes among Korean workers and examine potential effect modifications by working hours and shift work status.

**METHODS:**

This study analyzed data from the 2024 wave of the Korean Work, Sleep, and Health Study, a nationwide panel study of workers aged 19 years to 70 years (n=5,195). WTC was measured using a 6-item scale, and participants were categorized into low (≤12) or high (>12) WTC groups. Outcomes included self-rated health (SRH), depression (measured by the Patient Health Questionnaire-9), and work ability (WA; measured by the Work Ability Index). Logistic regression models were used to estimate odds ratios (ORs) and 95% confidence intervals (CIs) for poor SRH, moderate-to-severe depression, and poor WA, adjusting for demographic and occupational variables. Subgroup analyses assessed effect modifications by working hours and shift work.

**RESULTS:**

Higher WTC was associated with lower odds of poor SRH (OR, 0.73; 95% CI, 0.62 to 0.86), moderate-to-severe depression (OR, 0.71; 95% CI, 0.61 to 0.83), and poor WA (OR, 0.62; 95% CI, 0.46 to 0.84). These associations were significant among daytime workers but not shift workers. The protective effects of WTC were attenuated among workers whose working hours exceeding 52 hr/wk.

**CONCLUSIONS:**

Higher WTC is associated with better health and work outcomes, emphasizing its importance for employee well-being. However, its benefits may be limited among shift workers and employees working excessive hours.

## GRAPHICAL ABSTRACT

[Fig f1-epih-47-e2025036]

## Key Message

• Higher work-time control (WTC) was associated with better self-rated health, lower risk of depression, and improved work ability among Korean workers.

• The protective effects of WTC were significant for daytime workers but not for shift workers, and benefits diminished when weekly working hours exceeded 52.

## INTRODUCTION

Work-time control (WTC) refers to employees’ ability to influence their work schedules, including adjusting work hours, managing break times, and scheduling vacations [1]. As a key component of temporal job autonomy, WTC enables individuals to align work demands with personal and family needs, fostering improved work-life balance. The significance of WTC in modern workplaces has grown as organizational practices increasingly prioritize flexible work arrangements. WTC not only benefits employees by reducing stress and enhancing well-being but also supports organizations by improving productivity and lowering absenteeism [2,3].

A lack of WTC has been linked to adverse health outcomes, including higher rates of psychological distress, poorer self-rated health (SRH), and reduced work ability (WA). Employees with limited control over their schedules often experience greater work-life interference [4], insufficient recovery time, and elevated stress levels [5], which contribute to poorer health and job dissatisfaction [1]. Existing research supports the association between WTC and worker health. For instance, Albrecht et al. [4] demonstrated that WTC mitigates work-life interference, subsequently improving mental and musculoskeletal health. Ala-Mursula et al. [6] showed that high WTC reduces sickness absence and promotes SRH. In a systematic review, Nijp et al. [1] reported positive relationships between WTC, work-life balance, and job satisfaction, although they noted limitations regarding causality. Additionally, WTC moderates the effects of job strain and effort-reward imbalance on health outcomes, emphasizing its protective role against work-related stress [3]. Nevertheless, prior studies have often focused on specific populations, such as healthcare workers or municipal employees, limiting generalizability to the broader working population.

The labor culture of Korea is characterized by some of the longest working hours among Organization for Economic Cooperation and Development countries, as well as a high prevalence of shift work and rigid workplace hierarchies [7]. These contextual factors may amplify the effects of WTC on health outcomes, making Korea a particularly relevant setting for investigating the implications of work-time autonomy. Therefore, this study aimed to comprehensively evaluate the impact of WTC on health-related and work-related outcomes among diverse occupational groups in Korea. The hypotheses were that employees with high WTC would report better SRH, lower levels of depression, and higher WA. Additionally, the associations of WTC with these outcomes were expected to differ based on working hours and shift schedules, reflecting the contextual influence of job characteristics.

## MATERIALS AND METHODS

### Study population

Data were obtained from the Korean Work, Sleep, and Health Study (KWSHS), a nationwide panel study initiated in 2022. Stratified random sampling based on age and sex was used to invite panelists to participate via an online survey platform. Eligible participants were Korean wage workers aged 19 years to 70 years. Additional details regarding the sampling procedure have been previously described by Cho et al. [8]. The study was conducted twice per year from July 2022 to September 2024, for a total of 5 online surveys. The present analysis used data from the final survey, conducted in September 2024, which included assessments of WTC. The survey collected data on demographic characteristics (age, gender, and occupation), working hours, and shift work status; furthermore, it included items assessing WTC, SRH, depression, and WA. Self-employed individuals were excluded from this analysis due to their variable business hours and greater autonomy over scheduling. Respondents with incomplete responses to key survey items were also excluded. Of the 5,783 participants who responded to the September 2024 survey, 5,195 were included in the final analysis.

### Measurements

#### Independent variable: WTC

To define WTC, we referred to the work of Ala-Mursula et al. [2]. Respondents rated their level of influence over the following aspects of their working hours: (1) the starting and ending times of the workday, (2) taking breaks during the workday, (3) handling private matters during the workday, (4) scheduling work shifts, (5) scheduling vacations and paid days off, and (6) taking unpaid leave. Each item was rated on a 5-point Likert scale: 1 (very little), 2 (slightly), 3 (moderately), 4 (much), and 5 (very much). The total score, ranging from 6 to 30, was used to categorize WTC. Scores of ≤12 were classified as indicating low WTC, whereas scores above 12 were considered high WTC. The cut-off score of 12, corresponding to an average score of 2 across the 6 items, maximized the Youden Index [9] for SRH, depression, and WA. Moreover, when odds ratios (ORs) for poor SRH, depression, and poor WA were calculated using various cut-off scores, a WTC cut-off of 12 produced the lowest p-values.

#### Dependent variables: SRH, depression, and WA

SRH was assessed using the question, “How would you rate your overall health?” Respondents selected 1 of 5 options: very good, good, fair, poor, or very poor. Responses of “poor” or “very poor” were categorized as “poor” SRH, while all other responses were categorized as “good or fair” SRH.

Depression was measured using the Patient Health Questionnaire-9 (PHQ-9) [10], which has demonstrated high reliability in its Korean-translated version and has been widely used in various studies [11,12]. The questionnaire consists of 9 items evaluating symptoms over the past 2 weeks: (1) little interest or pleasure in doing things; (2) feeling down, depressed, or hopeless; (3) trouble falling or staying asleep, or sleeping too much; (4) feeling tired or having little energy; (5) poor appetite or overeating; (6) feeling bad about oneself or feeling like a failure; (7) trouble concentrating on tasks, such as reading the newspaper or watching television; (8) moving or speaking so slowly that others notice, or being especially fidgety or restless; and (9) thoughts of self-harm or being better off dead. Respondents rated each item on a Likert scale with possible responses of 0 (not at all), 1 (several days), 2 (more than half the days), and 3 (nearly every day). Total scores, calculated by summing the item responses, were classified as follows: no depression (0 to 4), mild depression (5 to 9), moderate depression (10 to 14), moderately severe depression (15 to 19), and severe depression (20 to 27). For this study, scores of 10 or higher were categorized as “moderate-to-severe depression,” while scores of 9 or lower were classified as “no or mild depression.”

WA was assessed using the Work Ability Index (WAI) [13], a widely used tool in clinical occupational health research. The WAI has been extensively validated internationally and has demonstrated high reliability in the Korean context [14]. The questionnaire used in this study evaluated multiple dimensions of WA, health status, and mental well-being and comprised 7 sections: (1) current WA; (2) WA in relation to job demands; (3) existing diseases and diagnoses; (4) work impairment; (5) days absent from work due to illness; (6) predicted WA for the next 2 years; and (7) reflections on mental capacity. The total WAI score was calculated by summing the points from all sections, with certain items weighted based on job demands (physical, mental, or both). Only physician-confirmed diagnoses were included in the scoring regarding diseases. Total scores ranged from 7 to 49 and were categorized into 4 groups: poor (7 to 27), moderate (28 to 36), good (37 to 43), and excellent (44 to 49). In this study, scores of 27 or below were classified as “poor” WA, while scores above 27 were categorized as “moderate-to-excellent” WA.

#### Other variables

Data on gender, age, occupation, weekly working hours, and shift work status were collected for subgroup analyses and used as adjustment covariates. Occupations were classified as blue-collar (agriculture, forestry, fishery, crafts, machine operation, assembly, and manual labor), pink-collar (service and sales workers), or white-collar (managers, professionals, and office workers). Working hours were assessed relative to Korea’s statutory maximum of 52 hr/wk; individuals exceeding this threshold were evaluated for potential risk [7]. Shift work status was determined based on the response to the question, “Do you primarily work during the daytime (6 a.m. to 6 p.m.)?” Respondents who answered “yes” were categorized as daytime workers, while those who answered “no” were categorized as shift workers.

### Statistical analysis

Baseline characteristics of the study population were analyzed and stratified by WTC. Logistic regression analysis was used to examine the associations between WTC and SRH, depression, and WA. ORs and 95% confidence intervals (CIs) for poor SRH, moderate-to-severe depression, and poor WA were calculated using 3 models: an unadjusted model, model 1 (adjusted for gender and age), and model 2 (adjusted for gender, age, and occupation). Additionally, we analyzed whether working hours and shift work status moderated the associations of WTC with SRH, depression, and WA. Subgroup analyses were conducted by working hours (≤52 vs. >52 hr/wk), shift work status, and occupation, with interaction effects evaluated using p-values from likelihood ratio tests. All subgroup analyses were adjusted for gender, age, and occupation. Statistical analyses were conducted using R version 4.4.0 (R Foundation for Statistical Computing, Vienna, Austria), with 2-tailed p-values less than 0.05 considered to indicate statistical significance.

### Ethics statement

The study was conducted in compliance with the Declaration of Helsinki. Informed consent was obtained from all participants, and anonymity and confidentiality were ensured. The Institutional Review Board of Dong-A University approved the study protocol (IRB No. 2-1040709-AB-N-01-202202-HR-017-06).

## RESULTS

Table 1 details the characteristics of the study population. Among workers with low WTC, the majority were women (57.1%), whereas men comprised a larger proportion (56.9%) of workers with high WTC. Gender, age, and occupational distributions differed significantly between the WTC groups. However, no substantial differences in working hours or shift work status were observed between the groups. In the low WTC group, 16.7% exhibited poor SRH compared to 12.8% in the high WTC group, representing a significant difference. Similarly, moderate-to-severe depression was more prevalent in the low WTC group (19.3%) than in the high WTC group (15.2%). Poor WA was also more common among workers with low WTC (5.1%) than among those with high WTC (3.1%).

Table 2 summarizes the results of the logistic regression analysis examining associations of WTC with SRH, depression, and WA. Workers with high WTC had approximately 27% lower odds of poor SRH than those with low WTC (unadjusted: OR, 0.73; 95% CI, 0.62 to 0.86; model 1: OR, 0.73; 95% CI, 0.61 to 0.86; model 2: OR, 0.73; 95% CI, 0.62 to 0.86). Similarly, the odds of moderate-to-severe depression were over 25% lower among workers with high WTC compared to those with low WTC (unadjusted: OR, 0.75; 95% CI, 0.64 to 0.87; model 1: OR, 0.71; 95% CI, 0.60, 0.83; model 2: OR, 0.71; 95% CI, 0.61 to 0.83). Additionally, the odds of having poor WA were over 37% lower among workers with high WTC compared to those with low WTC (unadjusted: OR, 0.59; 95% CI, 0.44 to 0.79; model 1: OR, 0.61; 95% CI, 0.45 to 0.82; model 2: OR, 0.62; 95% CI, 0.46 to 0.84).

Table 3 presents the results of subgroup and interaction analyses by working hours and shift work status. The associations shown in Table 2 were significant among workers who adhered to Korea’s statutory maximum weekly working hours (≤52 hr/wk) [15], but they were not significant among those working more than 52 hr/wk. Similarly, significant associations between WTC and SRH, depression, and WA were found among daytime workers but not among shift workers. Interaction analysis revealed that shift work status significantly impacted the relationship between WTC and WA, representing the only significant interaction. In occupation-stratified analyses, high WTC was significantly associated with better health outcomes across all three measures—SRH, depression, and WA—among white-collar workers. Conversely, among blue-collar workers, significant associations were limited to depression and WA, and no statistically significant associations were observed among pink-collar workers.

## DISCUSSION

The primary objective of this study was to investigate the relationship between WTC and various health outcomes. Our analysis revealed significant associations between WTC and SRH, depression, and WA. Specifically, higher WTC was positively associated with better SRH, lower levels of depression, and greater WA.

These findings strongly align with the broader body of literature, underscoring the critical role of employee WTC in mitigating the detrimental effects of job stress. Numerous studies have highlighted WTC as a key factor in promoting psychological well-being and reducing workplace stress. For instance, Ala-Mursula et al. [6] reported that employees with a higher WTC had a lower risk of sickness absence than those with a lower WTC in both women and men. Vahtera et al. [16] provided evidence that high WTC reduced the risk of disability retirement due to mental health disorders by 35% in women (HR, 0.65; 95% CI, 0.51 to 0.79) and 41% in men (HR, 0.59; 95% CI, 0.47 to 0.74). Nijp et al. [1] systematically reviewed 63 relevant papers from 53 studies on the association of WTC with factors including psychological distress and job satisfaction, noting moderate to strong evidence of a protective effect on mental health outcomes. Albrecht et al. [4] further quantified the mediating role of WTC in reducing work-life interference, showing significant reductions in depressive symptoms (OR, 0.65; 95% CI, 0.50 to 0.85) and musculoskeletal pain (OR, 0.71; 95% CI, 0.58 to 0.88) among workers with higher WTC. Additionally, Griep et al. [5] found that employees with low WTC combined with long working hours faced a higher risk of stress (OR, 1.56; 95% CI, 1.11 to 2.20) and poor SRH (OR, 1.64; 95% CI, 1.13 to 2.38). While informative, these prior studies largely focused on specific occupations or public-sector contexts. In contrast, the present study utilizes a nationwide sample of Korean workers across various industries, thus offering broader generalizability and reflecting the heterogeneity of modern labor markets.

Korea’s unique cultural and organizational context may further amplify or modify these established relationships. Korean work culture is characterized by long working hours, rigid hierarchical structures, and collectivist values, all of which can exacerbate work-related stress [7]. Additionally, the high prevalence of shift work and extended working hours in Korea could increase pressure, making WTC particularly influential in this context. Our analysis indicates that even with high levels of WTC, the risk of poor WA remains elevated under conditions of long working hours or shift work. This suggests that the interaction between physical and psychological stress reduces the protective effects of WTC, especially in situations involving persistent overwork and irregular schedules [17]. However, the small sample sizes in these subgroups may have limited statistical power; thus, the results should be interpreted cautiously.

Furthermore, our stratified analysis revealed that the association between WTC and health outcomes varied by occupation. The benefits of WTC were most pronounced among white-collar workers, partially evident among blue-collar workers, and insignificant among pink-collar workers. These differences may result from limited flexibility in autonomously adjusting working hours among employees on production lines and service sectors [18]. Collectively, these findings suggest that although WTC consistently shows protective effects across diverse contexts, the magnitude and mechanisms of these effects are influenced by cultural, demographic, and occupational factors.

Several theoretical mechanisms may explain how WTC contributes to improved health outcomes. First, from the perspective of the effort-recovery model, WTC enables employees to manage work demands more flexibly, facilitating better recovery during and after work hours [19]. This can reduce cumulative fatigue and stress, which are known risk factors for poor physical and mental health. Second, according to the job demands-resources model, WTC is a key resource providing workers with greater autonomy and control—factors that buffer against the adverse effects of job strain and psychological distress [20,21]. Third, WTC may improve individuals’ work-life balance, which is strongly linked to better SRH and lower depression risk [22]. Moreover, greater scheduling control can facilitate engagement in and access to health-promoting behaviors, such as regular sleep, physical activity, and timely medical care [23].

Several limitations of this study should be acknowledged. First, its cross-sectional design precludes the establishment of causal relationships between WTC and observed health-related and work-related outcomes because the temporal sequence of these variables remains unclear. Future longitudinal or interventional studies are warranted to clarify the causal pathways and confirm the associations observed in this study. Second, reliance on self-reported data might introduce biases such as social desirability or recall bias, potentially affecting the accuracy of the findings [24,25]. Finally, the study’s focus on Korea’s specific cultural and organizational context limits the generalizability of the results to other populations or work environments with differing cultural, economic, and occupational characteristics.

In conclusion, our findings build upon previous evidence demonstrating the critical role of WTC in improving health-related and work-related outcomes. From a policy perspective, our results emphasize the need for organizational interventions to promote WTC. Flexible work arrangements—particularly those supporting employee-directed flexibility such as employee-directed flex time and self-scheduling—can improve employee well-being, WA, and productivity [26]. It is important to distinguish these from employer-based flexibility, which may involve unpredictable scheduling and has been associated with adverse health outcomes in previous research [27,28]. Policies tailored to specific groups, such as employees with long working hours and/or shift work schedules, may yield additional benefits, since these groups are often more vulnerable to the adverse effects of rigid work schedules. Implementing these policies can foster healthier and more inclusive workplaces, while identifying the contextual factors influencing these relationships provides valuable insights for organizations aiming to improve worker well-being. Future research should focus on diverse populations and (quasi-)experimental designs to establish WTC as a cornerstone of occupational health interventions.

## Figures and Tables

**Figure f1-epih-47-e2025036:**
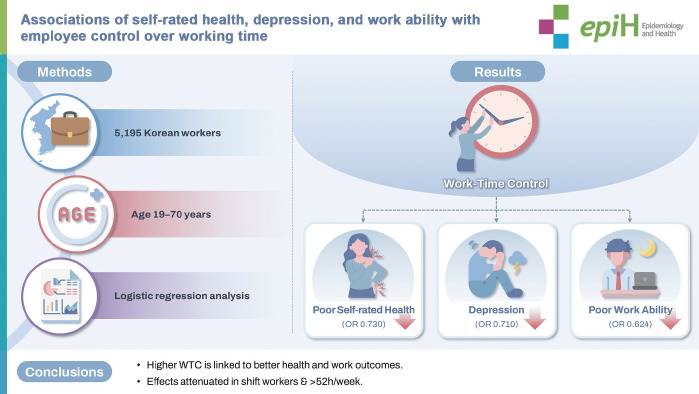


**Table 1.** Baseline characteristics of the study population

Characteristics	Overall (n)	Work-time control		
		Low	High	p-value
Total (n)	5,195	1,561 (30.0)	3,634 (70.0)	
Gender				<0.001
Men	2,739	670 (24.5)	2,069 (75.5)	
Women	2,456	891 (36.3)	1,565 (63.7)	
Age (yr)				<0.001
20-29	884	219 (24.8)	665 (75.2)	
30-39	1,115	300 (26.9)	815 (73.1)	
40-49	1,240	388 (31.3)	852 (68.7)	
50-59	1,253	433 (34.6)	820 (65.4)	
≥60	703	221 (31.4)	482 (68.6)	
Occupation				<0.001
Blue collar	797	278 (34.9)	519 (65.1)	
Pink collar	416	142 (34.1)	274 (65.9)	
White collar	3,982	1,141 (28.7)	2,841 (71.3)	
Working hours (hr/wk)				0.160
≤52	4,953	1,478 (29.8)	3,475 (70.2)	
>52	242	83 (34.3)	159 (65.7)	
Shift work status				0.266
Daytime worker	4,713	1,405 (29.8)	3,308 (70.2)	
Shift worker	482	156 (32.4)	326 (67.6)	
Self-rated health				<0.001
Good or fair	4,470	1,300 (29.1)	3,170 (70.9)	
Poor	725	261 (36.0)	464 (64.0)	
Depression				<0.001
No or mild depression	4,339	1,259 (29.0)	3,080 (71.0)	
Moderate-to-severe depression	856	302 (35.3)	554 (64.7)	
Work ability				<0.001
Moderate-to-excellent	5,003	1,481 (29.6)	3,522 (70.4)	
Poor	192	80 (41.7)	112 (58.3)	

Values are presented as number (%).

**Table 2.** Self-rated health, depression, and work ability for employees with high (relative to low) work-time control^1^

Variables	Self-rated health: poor	Depression: moderate-to-severe	Work ability: poor
Unadjusted	0.73 (0.62, 0.86)^***^	0.75 (0.64, 0.87)^***^	0.59 (0.44, 0.79)^***^
Model 1	0.73 (0.61, 0.86)^***^	0.71 (0.60, 0.83)^***^	0.61 (0.45, 0.82)^**^
Model 2	0.73 (0.62, 0.86)^***^	0.71 (0.61, 0.83)^***^	0.62 (0.46, 0.84)^**^

Values are presented as odds ratio (95% confidence interval).

1 Model 1 is adjusted for gender and age; Model 2 is adjusted for gender, age, and occupation.

** p<0.01,

*** p<0.001.

**Table 3.** Associations of self-rated health, depression, and work ability with high (relative to low) work-time control, stratified by working hours, shift work status, and occupation

Variables	Self-rated health: poor	Depression: moderate to severe	Work ability: poor
Working hours (hr/wk)^1^			
≤52	0.73 (0.61, 0.87)^***^	0.71 (0.60, 0.84)^***^	0.60 (0.44, 0.81)^**^
>52	0.82 (0.40, 1.64)	0.73 (0.39, 1.37)	1.48 (0.38, 5.82)
Interaction p-value	0.697	0.991	0.215
Shift work status^1^			
Daytime worker	0.72 (0.61, 0.86)^***^	0.71 (0.60, 0.84)^***^	0.55 (0.40, 0.76)^***^
Shift worker	0.74 (0.42, 1.31)	0.76 (0.45, 1.28)	1.54 (0.59, 4.04)
Interaction p-value	0.728	0.851	0.041
Occupation^2^			
Blue collar	0.81 (0.53, 1.23)	0.59 (0.40, 0.89)^*^	0.51 (0.26, 1.00)^*^
Pink collar	0.61 (0.36, 1.04)	0.74 (0.43, 1.27)	0.80 (0.33, 1.94)
White collar	0.73 (0.60, 0.89)^**^	0.73 (0.61, 0.88)^**^	0.63 (0.44, 0.90)^*^
Interaction p-value	0.704	0.534	0.729

Values are presented as odds ratio (95% confidence interval).

1 Adjusted for gender, age, and occupation.

2 Adjusted for gender and age.

* p<0.05,

** p<0.01,

*** p<0.001.
